# Neurophysiology of movement inhibition during full body reaching

**DOI:** 10.1038/s41598-022-18677-9

**Published:** 2022-09-16

**Authors:** Rachel L. M. Ho, Wei-en Wang, Susanne M. van der Veen, Ajay Antony, James S. Thomas, Stephen A. Coombes

**Affiliations:** 1 Laboratory for Rehabilitation Neuroscience, Department of Applied Physiology and Kinesiology, University of Florida, P.O. Box 118205, Gainesville, FL 32611-8205 USA; 2 The Orthopedic Institute, 4500 W Newberry Road, Gainesville, FL 32607 USA; 3 Department of Physical Therapy, Virginia Commonwealth University, VCU College of Health Professions, 900 E. Leigh, Street Box 980233, Richmond, VA 23298 USA

**Keywords:** Motor control, Musculoskeletal system

## Abstract

Our current understanding of response inhibition comes from go/no-go studies that draw conclusions based on the overt movement of single limbs (i.e., a single finger pushing a button). In general, go/no-go paradigms have found that an individual’s ability to correctly inhibit the motor system is indicative of a healthy central nervous system. However, measuring inhibition by an overt behavioral response may lack the sensitivity to conclude whether the motor system is completely inhibited. Therefore, our goal was to use behavioral and neurophysiological measures to investigate inhibition of the motor system during a full-body reaching task. When directly comparing neurophysiological and behavioral measures, we found that neurophysiological measures were associated with a greater number of errors during no-go trials and faster onset times during go trials. Further analyses revealed a negative correlation between errors and onset times, such that the muscles that activated the earliest during go trials also had the greatest number of errors during no-go trials. Together, our observations show that the absence of an overt behavioral response does not always translate to total inhibition of the motor system.

## Introduction

Much of our understanding of response inhibition in humans is derived from go/no-go experiments. First published in 1969, go/no-go paradigms require voluntary movement to a go stimulus (i.e. push a button when a light turns green) and inhibition of movement to a no-go stimulus (i.e. do nothing when a light turns red)^1,2^. Narrow response windows promote faster movements, thus priming the motor system for action, and making it harder to correctly inhibit movement. Conventional studies have been limited to single limb movements (i.e., finger movement) and drawn conclusions on response inhibition based on the presence or absence of an overt behavioral response (i.e., button press)^3–6^. The goal in the current study was to extend our understanding of movement inhibition in humans by implementing a go/no-go paradigm within a full body reaching task while measuring activation of the motor system at the behavioral and neurophysiological level.

Go-no/go paradigms have been widely used to investigate response inhibition in healthy adults^7,8^, adolescents^9,10^, as well as individuals with a range of disorders^11–15^. Using go/no-go button press tasks, these studies have generally found that response inhibition is compromised in varying disorders, and that one’s ability to inhibit the motor system is a fundamental component of a healthy central nervous system. For example, individuals with higher levels of impulsivity and lower IQ incorrectly respond to no-go stimuli (termed errors of commission) more often than those with lower levels of impulsivity and higher IQ^11^. However, an important caveat to using overt motor responses is that the absence of an error of commission does not necessarily translate to complete inhibition of the motor system at the neurophysiological level. Indeed, not only are there differing levels of sensitivity in response devices (how much force does one need to apply for a response to be registered) but brain and muscle can activate without resulting in an overt behavioral response^16^. To our knowledge, no one has implemented a go/no-go paradigm within a full body reaching movement and used neurophysiological measures, in combination with behavioral measures to quantify errors of commission.

In the current study, we implemented our full-body reaching task^17^ within a novel go/no-go experimental paradigm. We developed this task in virtual reality (VR) space and combined it with electromyography (EMG) measurements. Virtual targets were standardized such that participants could in theory reach the target during go trials by flexing their lumbar spine 15° (high target) or 60° (low target) with the shoulder flexed 90° and the elbow fully extended. In addition to measuring movement of the hand controller, which tracked hand position, (i.e., an overt behavioral response) we also measured muscle activity from the tibialis anterior, deltoid and multifidus muscles. We first hypothesized that during no-go trials, errors of commission would be greater when calculated using neurophysiology measures (muscle activity measured by EMGs) as compared to an overt behavioral measure (hand position). Second, we hypothesized that onset times would be faster using neurophysiology measures as compared to behavioral measures, and that higher errors of commission during no-go trials would be associated with faster onset times during go trials. Finally, given the further distance of the low target, we expected errors of commission to be greater and onset times to be faster for low as compared to high targets.

## Results

### No-go errors of commission analysis across EMGs and hand position

**Figure 1:**
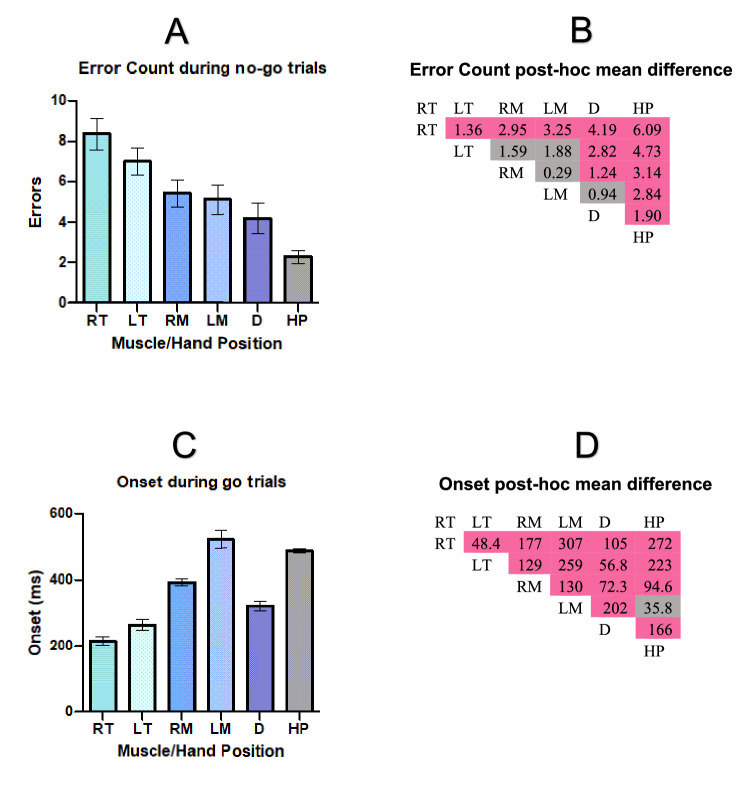
Results from errors of commission analysis across no-go trials and onset analysis across go trials. (**A**,**B**) show the results of the errors of commission analysis during no-go trials. In general, (**A**) demonstrates that hand position had significantly less errors than all the muscles we investigated. (**B**) shows the mean difference for errors of commission between muscles and hand position. Color demonstrates level of significance with grey indicating a p-value > 0.05, and dark pink indicating a p-value < 0.01. (**C**,**D**) show the results of the onset analysis across go trials. In general, (**C**) shows that onset time was significantly slower for hand position than all the muscles we investigated, except the left multifidus. (**D**) shows the mean difference for onset between muscles and hand position. *RT* right tibialis, *LT* left tibialis, *RM* right multifidus, *LM* left multifidus, *D* right deltoid, *HP* hand position.

We hypothesized that during no-go trials errors of commission would be greater when calculated using neurophysiology measures as compared to an overt behavioral response. Results from our two-way, repeated-measure ANOVA found a significant effect of height (p = 0.045) and a significant effect of muscle/hand position (p = 0.008). No significant interactions were found (height × sex = 0.397, muscle/hand position × sex = 0.222, height × muscle/hand position = 0.443, height × muscle/hand position × sex = 0.285). Post-hoc analysis for the main effect of height indicated that more errors were committed at the low than high target (high target mean and standard deviation: 4.9 ± 0.44, low target mean and standard deviation: 5.9 ± 0.51). Data for errors of commission on the main effect of muscle/hand position are shown in Fig. 1A and means and standard errors are presented in Table 1. For ease of interpretation, error count is collapsed across target height. Results from FDR corrected post-hoc analysis can be found in Fig. 1B. Grey color indicates a p-value > 0.05, while pink color indicates a p-value < 0.01. We found that errors of commission in right tibialis anterior were highest and significantly greater than the errors of commission in left tibialis anterior, right multifidus, left multifidus, deltoid, and hand position. Errors of commission in the left tibialis anterior were not significantly greater than right multifidus and left multifidus but were greater than the deltoid and hand position. There were more errors of commission committed in the right multifidus than the deltoid and hand position and in the left multifidus compared to hand position, but the two multifidi muscles were not statistically different from one another. Notably, errors of commission in hand position were significantly lower than all other measures, suggesting attenuated sensitivity to errors in behavioral as compared to neurophysiological measures.

**Table 1:** CE mean and standard error and onset mean and standard error.

	CE		ONSET	
	Mean	SE	Mean	SE
RT	8.36	0.78	215.16	11.81
LT	7.00	0.66	263.56	16.85
RM	5.41	0.69	392.75	9.94
LM	5.12	0.73	523.14	26.98
D	4.18	0.73	320.36	14.88
HP	2.28	0.32	487.31	4.84

*CE* errors of commission, *RT* right tibialis anterior, *LT* left tibialis anterior, *RM* right multifidus, *LM* left multifidus, *D* deltoid, *HP* hand position.

### Onset analysis across EMGs and hand controller

We hypothesized that onset times would be faster using neurophysiology measures as compared to behavioral measures. The results from our two-way, repeated-measure ANOVA found an effect of muscle/hand position (p < 0.001), but no effect of height (p = 0.204) or interaction (height × sex = 0.204, muscle/hand position × sex = 0.272, height × muscle/hand position = 0.896, height × muscle/hand position × sex = 0.556). See Table 1 for onset means and standard errors and Fig. 1D for results from the FDR corrected post-hoc analysis. Onset times for each muscle/hand position are shown in Fig. 1C. Onset times were fastest in right tibialis anterior and slowest in the left multifidus. Notably, all onset times were statistically different from each other except between the left multifidus and hand position.

### Correlation between errors of commission and onset across all measures

**Figure 2:**
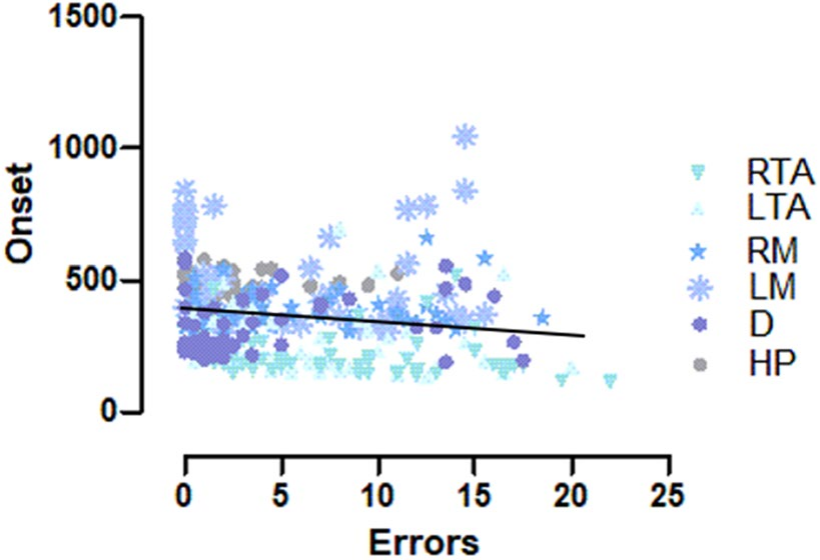
Relationship between errors of commission and onset. Each symbol on the scatter plot is indicative of an individual’s mean error of commission and mean onset. The overall trend of the scatter plots shows a negative correlation with higher errors of commission during no-go trials being associated with faster onsets during go trials.

Figure 2 shows the relationship between errors of commission during no-go trials and onset times during go trials across all measures. There was a significant negative correlation between the two variables, r = − 0.221, p < 0.001 with higher errors of commission associated with faster onset times. All correlations for each muscle/hand position failed to reach significance (all p’s > 0.05).

## Discussion

The goal of the current study was to determine whether errors of commission derived from behavioral and neurophysiology measures differ during a go/no-go full body reaching task. Our study is the first to successfully combine a go/no-go paradigm with a standardized full body reaching task in virtual reality space. Our observations show that when directly comparing neurophysiological and behavioral measures, neurophysiological measures were associated with a greater number of errors during no-go trials and faster onset times during go trials. Further analyses revealed a negative correlation between errors and onset times when all the measures were combined together (muscles + hand placement), such that the muscles that activated the fastest during go trials also had the greatest number of errors during no-go trials. Errors and onset times also followed a distal to proximal cascade consistent with evidence of anticipatory postural adjustments (APAs) in reaching tasks^18^.

Previous studies have used EMG to examine the neural mechanisms underlying movement inhibition, during both go/no-go and stop signal tasks^4,8,19^. Although a partial response EMG can be evident during successful no-go trials and successful stop signal trials, the mechanisms underlying each process is different^16,20^. No-go trials require action restraint, where stop trials require action cancellation. Here we focus on action restraint. Whereas previous studies have used a button press as the overt behavioral response and EMG recordings from a single muscle, we implemented a full body reaching task, recorded EMG from multiple muscles, and compared number of errors of commission across behavioral and neurophysiological measures. Compared to an overt behavioral response, errors of commission were higher when using muscle activity as a readout of motor system activation. Our behavioral measure of hand position had significantly less errors compared to all neurophysiology measures. Errors of commission for overt behavioral responses on no-go trials was 4.38% in the current study, which is consistent with values of ~ 5% reported in studies using index finger key press tasks^21,22^. Error rates rose to 8–16% when using EMG measures in our study, in line with findings by Raud et al.^16^ who reported EMG activity in the abductor pollicis brevis during 14% of successful no-go trials in which there was no overt button press. Together these findings suggest that despite different tasks (full body reaching vs button press), and recordings from different effectors (finger vs torso vs limbs), responses during no-go trials are remarkably similar, which points to a relatively stable movement inhibition mechanism in humans.

Although recent evidence points to clear differences between the spatial and temporal profiles of go-no/go tasks and stop signal tasks^16^, a fundamental role of prefrontal cortex in movement inhibition is well established^5,23–27^. Lesions in prefrontal cortex are associated with worst performance on tasks that require response inhibition, as compared to tasks that do not^5,27^. Removing a bilateral meningioma tumor in medial prefrontal cortex led errors of commission to drop from 75% to normal levels^26^, providing causal evidence for the association between medial prefrontal cortex function and movement inhibition during a go-no/go task. Other support comes from in-vivo neuroimaging studies^23–25^. Konishi et al.^25^ found consistent brain activation in right inferior prefrontal cortex irrespective of which hand completed the go/no-go task suggesting effector independence in the prefrontal inhibitory mechanism. This is important because it suggests that the effector being used, and its corresponding somatotopic organization in the human motor system, does not appear to influence how easy or difficult it is to restrain action.

Errors of commission were higher for more distal as compared to proximal muscles. For instance, the largest number of errors were evident in the right and left tibialis anterior, and the fewest in the deltoid muscle. APAs during reaching movements is one explanation for this pattern in the data^18,28–30^. APAs reflect a robust and consistent adjustment in posture that is made in anticipation of the arm moving away from the center of mass, and characterized by activity in distal musculature like the tibialis and gastrocnemius muscles prior to activation of muscle in the torso and arm^18,29–33^. Other research has investigated how the neural control of APAs is effected by aging^34–37^, pain^38–41^, Parkinson’s Disease^42,43^, and stroke^44,45^. For example, APAs during arm movements in the elderly are characterized by slower onset latencies in the tibialis anterior and gastrocnemius^36^ and by a hip strategy that promotes stability by recruiting muscle activity in a more proximal to distal manner^35^. We found the opposite pattern of activity in the current study in young healthy adults, with errors of commission occurring more often in distal as compared to proximal muscles. Future research utilizing EMGs as a neurophysiological measure of inhibition can determine how age and disease may modulate this pattern during full body reaching.

Higher errors of commission during no-go trials were associated with faster onsets during go-trials. Our overt behavioral measure of hand position occurred with an average onset time of 487 ms and this onset was greater than the onset of activity in all of the muscles except the left multifidus. Individual correlations at each muscle/hand position failed to reach significance. This means that the association between onset time and CEs only emerges when combining data across multiple muscles. This relationship suggests future research implementing a neurophysiological method of measuring response inhibition should consider the typical cascade of onset of muscle activity associated with their task, as our results suggest that muscles with the fastest onset are the same muscles that will commit the most errors of commission during response inhibition. Together, our results suggest that the lack of an overt behavioral response is not sufficient to assume that the motor system is completely inhibited. Conclusions on response inhibition based on go/no-go paradigms that only measure overt movements should be made with caution, given that muscle can be active despite no evidence of an overt behavioral response.

## Methods

### Participants

The University of Florida Institutional Review Board approved this study and research adhered to relevant guidelines and regulations. Informed consent was collected from all subjects prior to data collection. All data collection occurred at the University of Florida, and we recruited 51 participants for this study. Exclusion criteria for all participants included self-reported history of Parkinson’s disease, Alzheimer’s disease, multiple sclerosis, amyotrophic lateral sclerosis, TIA, stroke, seizures, epilepsy, cancer, heart disease, pregnant. The mean age was 19.5 years (± 0.72), 14 were male and 38 were female.

### Equipment

A Delsys Trigno Wireless system (Delsys Inc., Boston MA, USA) was used to collect surface EMG from seven muscles. One electrode was placed over the right deltoid (Delt) to quantify movement of the right arm during reaching. We placed EMGs over the right and left tibialis anterior (RTA, LTA) given their role in anticipatory postural adjustments during forward displacement of the torso^46^. To represent the erector spinae group^47,48^ we placed electrodes over the right and left multifidus (RMulti and LMulti). We did not collect data from the biceps and triceps brachii as we were not anticipating strong involvement of elbow flexion and extension due to participants starting each trial standing with upright posture, and their arm relaxed by their side with their elbow fully extended. EMG sampling rate was 1000 Hz.

An HTC VIVE virtual reality system (HTC Corp., New Taipei City, Taiwan) was used to project the game space. The game was programed using the Unity Virtual Reality platform (Unity Technologies, Copenhagen, Denmark). Hardware included the VIVE headset, two wireless hand controllers to track position of the hand, and two base stations for continuous localization of the headset, controllers, and trackers in space. The virtual reality game space was projected to the participant’s headset as well as a 30″ computer monitor (Dell UltraSharp U3011, Dell Co, Round Rock, TX) to ensure that the researcher could see what the participant was seeing within the VR environment. A Motion Monitor (Innovative Sports Training, Inc., Chicago, IL) system and an Arduino microcontroller (Arduino LLC., Italy) was used to synchronize data from the VR equipment and the EMG electrodes. Accuracy of position and orientation of the VIVE trackers/controllers is within 0.68 ± 0.32 cm translationally, and 1.64 ± 0.18° rotationally^11^. Sampling rate of VIVE controllers was 103 Hz.

### Calibration of target height and virtual reality avatar

Target locations were calculated from anthropometric measurements taken from each participant using one VR hand controller that marked the following locations on the body: the ground directly between the participant’s medial malleoli, top of the head, C-7, L-5, right greater trochanter, right acromioclavicular joint, right elbow, and tip of the right middle finger. From these coordinate data, target location in the VR space was normalized to each participant’s anthropometric measurements (i.e., hip height, trunk length, and arm length; see^49^ for more details). Thus, the virtual targets were located such that the participants could reach the target, in theory, by flexing their hips 15 degrees (high target) and 60 degrees (low target), with the shoulder flexed 90 degrees and the elbow fully extended. The coordinate data were also used to scale the participant’s avatar in VR space to match real-world coordinates to ensure fidelity of visual feedback in the VR space.

### Virtual reality reaching task

**Figure 3:**
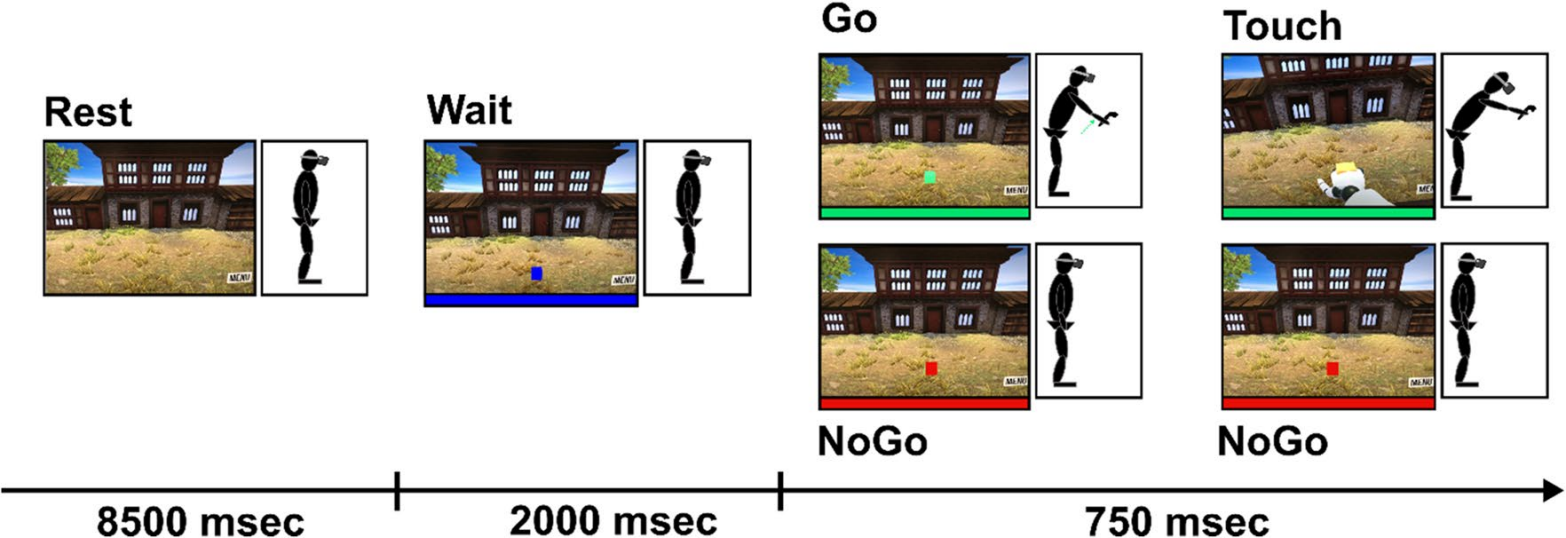
Individual trial of virtual reality-reaching task. Each square shows the visual display that the participant viewed in the VR environment. Cartoon figures reflect an example of subject posture during each phase of the trial. Participants started each trial with upright posture with both hands hanging at their side. After 8500 ms of rest, a blue cube appeared and represented the target they would reach out and touch. After 2000 ms, the same cube turned green or red. Participants were instructed to reach to the target if it turned green or stand still if it turned red. The red/green target remained on the screen for 750 ms. The blue, green and red bars under each square correspond with the blue, green, and red target cube. These bars were not visible to the participant.

Participants started each trial with upright posture and each hand holding a VR controller relaxed at their sides. Figure 3 shows the timeline, visual display, and cartoon of body position during a single virtual reality reaching trial. Each trial began with a rest period. Next, participants were told that a blue cube would appear in front of them and that this cube represents the target they need to reach forward and touch. After 2000 ms, the same cube turned green (go trial) or red (no-go trial). The participant was told to stand still if the cube turned red. If the cube turned green, the participant reached out with their right hand and touched the cube. The cube remained green or red for 750 ms and participants were instructed to reach and touch the green cube before it disappeared. Once the target disappeared participants had 8.5 s to return to the starting position and get ready for the next trial. Participants were told to only use their right hand when reaching for a green cube. Therefore, data analysis was only performed on the right-hand controller and EMG was placed on the right deltoid and not the left. Participants were not given instructions on how to move to avoid biasing natural movements. Participants were informed to move “in any manner that allowed for reaching the target as long as their feet remained stationary”.

### Experimental protocol

EMG electrodes were first attached to the participant. Placement sites were determined through palpation and then cleaned with alcohol wipes. EMGs were taped in place to prevent shifting. Next, participants were asked to stand facing an empty wall in the data collection space while the VR headset was placed on top of their head and over their eyes. Once the VR headset was secure, participants received instruction about the go/no-go task. One block consisted of 26 reaching trials and participants completed 4 blocks in total (i.e., 104 trials in total). The target was placed at a height that in principle could elicit 15° of lumbar flexion (high target) for two blocks and 60° of lumbar flexion (low target) for two blocks. Participants cycled through the four blocks beginning at either the high or the low target height, followed by the opposite target height (H–L–H–L or L–H–L–H). The starting target height was counterbalanced across participants. Additionally, the VR task was programmed to deliver half of the trials in each block as a go stimulus and half of the trials in each block as a no-go stimulus. The order of go and no-go trials within a block was random. A total of 13 go trials and 13 no-go trials were provided in each 26-trial block. A practice session of 10 trials (5 go, 5 no-go) at the high target and 10 trials at the low target were provided to each participant before data collection began.

### Data analysis

A customized Matlab program was used to analyze position of the right hand controller data. First, data was low pass filtered at 20 Hz. Second, a “distance window” was set to locate the max distance the hand traveled. This window started 1 ms after the target changed color and ended 1 ms before the end of the trial. Third, a “baseline window’ was set for locating the mean and standard deviation of the signal during the baseline, which was used to normalize overall signal. The window started 90 ms after the blue target appeared and ended 20 ms later. Once the signal was normalized using the baseline mean, the max distance the hand traveled and onset of the movement of the hand was calculated. The max distance was located within the distance window. Onset was calculated as the point when the signal increased by 5% of the max distance traveled. Trials with anticipatory activity were not included in the analysis. A threshold was set to determine if the hand moved during no-go trials. Based on position and orientation accuracy of VIVE trackers/controllers^50^ our threshold for a commission error was 0.68 cm + three times the standard deviation 0.32 cm. If the max distance the hand traveled during no-go trials passed this threshold the trial was marked as an error of commission, meaning the hand moved in response to a no-go stimulus. A summary statistic was calculated by summing the number of errors of commission that occurred on no-go trials for each individual and for each target height.

A customized Matlab program was used to analyze EMG data. First, data was high-pass filtered at 2 Hz (Butterworth 4th order dual pass), rectified, and low-passed filtered at 6 Hz (Butterworth 4th order dual pass). These filter settings were used to ensure consistent identification of onset of bursting of EMG activity^51,52^. Second, a window was set for locating peak amplitude and onset of EMG activity. The window started after the target changed color from blue to red or green and ended with termination of the trial. This ensured that anticipatory muscle activity was not included in determining peak amplitude and onset. Third, onset and peak EMG amplitude was calculated for all go trials. Peak amplitude was calculated as the maximum value that occurred between target color change and termination of the trial. The maximum value was normalized to the average EMG activity during the baseline window. The baseline window consisted of 1000 ms starting when the target turned blue. We calculated onset as the point when EMG activity increased by ≥ 5% of the normalized peak amplitude. Average peak amplitude was calculated for each muscle at both the high and low target during the go trials.

**Figure 4:**
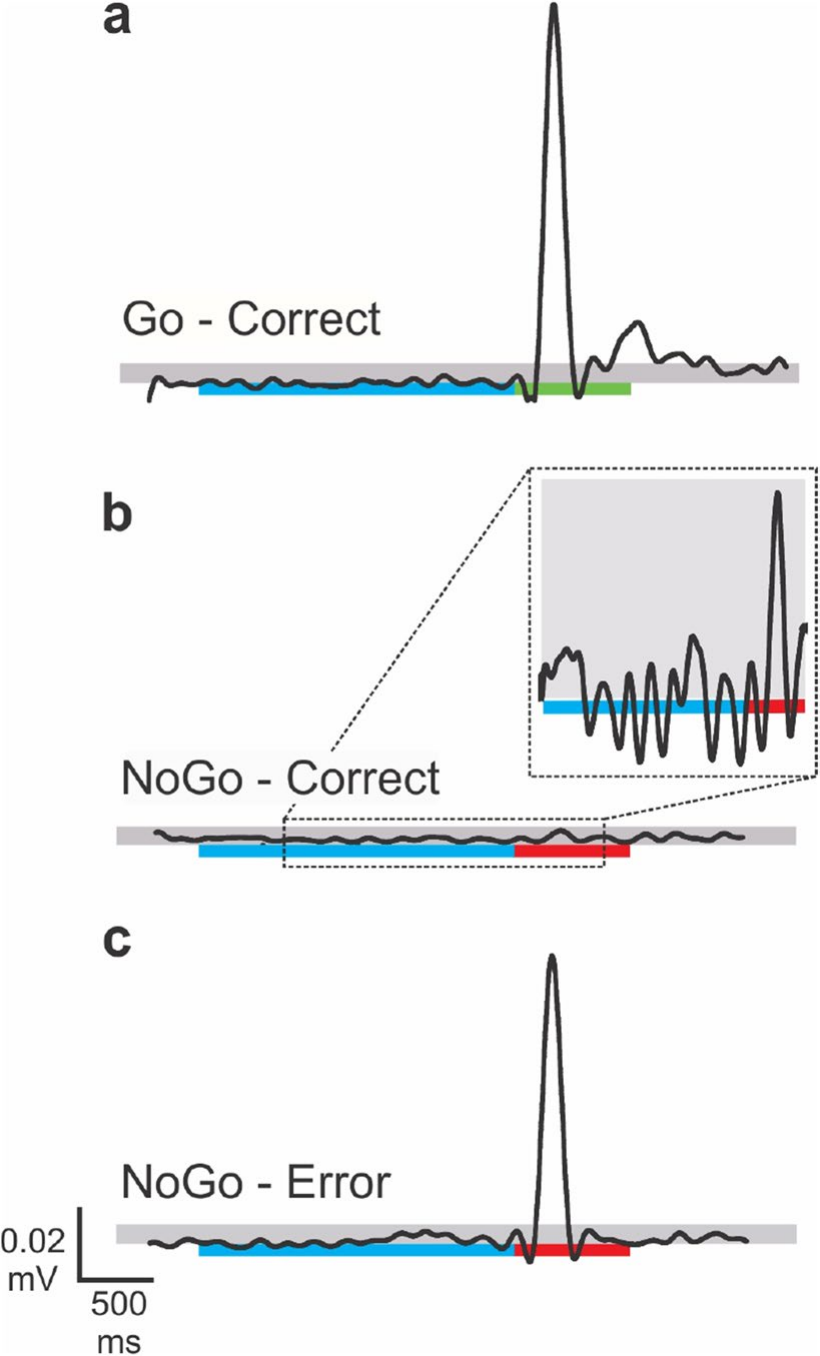
EMG threshold detection. Example EMG activity (black line) is shown during a go trial (**a**), a no-go trial with no error of commission (**b**), and a no-go trial with a error of commission (**c**). The correct response to a no-go trial was to stand completely still when the red cube appeared. An error during a no-go trial was the production of muscle activity in response to the appearance of the red cube. The blue, green, and red bars represent the appearance of the blue, green, and red cube. The grey shading represents the threshold. Trial “b” is labelled as correct because the peak amplitude does not cross the threshold, while trial “c” is labelled as an error because the peak amplitude crosses the threshold.

We used a threshold to quantify the extent of muscle activity produced during no-go trials. The threshold was calculated as the average baseline EMG activity plus 3 × the standard deviation. Each threshold was calculated individually for each participant and for each muscle. If the EMG activity during a no-go trial passed the threshold, the trial was labeled as an error of commission. Finally, all trials were plotted for visual inspection to ensure that our automated analysis approach was correctly performed. Figure 4 shows three example EMG time series from three different trials. The black lines represent the filtered EMG signal. The blue, green, and red bars below the time series represent the color of the target across time. The grey shading behind the EMG time series demarcate the amplitude of the threshold. Figure 4a shows a go trial that was labelled as correct. Figure 4b shows a no-go trial that was also labelled as a correct response because the EMG signal did not rise above the threshold. Figure 4c shows EMG activity during a no-go trial which was identified as a commission error because the peak amplitude passed the set threshold. The EMG response in Fig. 4a,c was therefore similar, but one was labelled as an error and one was labeled as a correct response based on the trial type (i.e., go vs no-go). The summary statistic was calculated by summing the number of errors of commission that occurred on no-go trials for each individual, for each muscle, and for each target height.

### Statistical analysis

To determine whether errors of commission were greater and onset times were faster for neurophysiological as compared to behavioral measures we ran separate two-way, repeated-measure ANOVAs with height (high target, low target) and muscle/hand position (5 EMGs, 1 hand controller) as independent variables, with sex as a covariate. Significant main effects and interactions were followed up with separate, repeated measure ANOVAs at the high and low target and t-tests as appropriate. All follow-up tests were corrected for multiple comparisons using FDR correction^53^. To explore the relationship between errors of commission during no-go trials and onset times during go trials we found the average (across target heights) of each individual’s error of commission and onset. This data was used to produce a Pearson Product Correlation at each muscle/hand position as well as a Person Product Correlation across all the measures. All analyses were performed in IBM SPSS Statistics for Window, version 25 (IBM Corp., Armonk, N.Y., USA).

## Data Availability

The datasets generated and/or analyzed during this study are not publicly available because participants of this study did not agree for their data to be shared publicly but are available from the corresponding author on reasonable request.
